# Circular Polarization Conversion in Single Plasmonic Spherical Particles

**DOI:** 10.1021/acs.nanolett.1c03848

**Published:** 2022-02-03

**Authors:** Pritam Khan, Grace Brennan, Zhe Li, Luluh Al Hassan, Daragh Rice, Matthew Gleeson, Aladin A. Mani, Syed A. M. Tofail, Hongxing Xu, Ning Liu, Christophe Silien

**Affiliations:** † Department of Physics and Bernal Institute, University of Limerick, Limerick V94 T9PX, Ireland; ‡ Department of Chemical Sciences and Bernal Institute, University of Limerick, Limerick V94 T9PX, Ireland; § School of Physics and Technology, Institute for Advanced Studies and Center for Nanoscience and Nanotechnology, Wuhan University, Wuhan, 430072, China

**Keywords:** Plasmon, damping, dark-field microscopy, polarization, adsorption, nanoporous, particles, polarization conversion, molecule, sensing, photon spin angular momentum, spin−orbit interaction

## Abstract

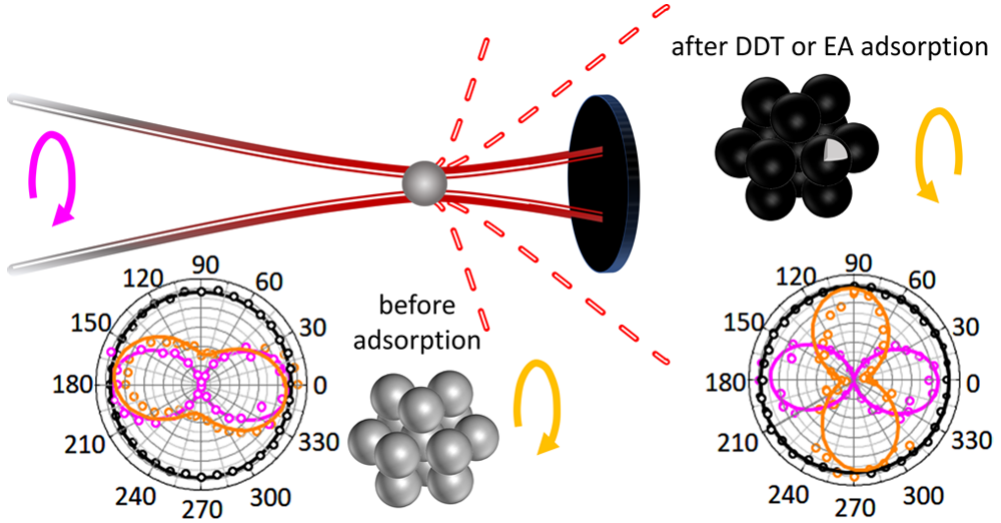

Temporal and spectral behaviors of plasmons determine their ability to enhance the characteristics of metamaterials tailored to a wide range of applications, including electric-field enhancement, hot-electron injection, sensing, as well as polarization and angular momentum manipulation. We report a dark-field (DF) polarimetry experiment on single particles with incident circularly polarized light in which gold nanoparticles scatter with opposite handedness at visible wavelengths. Remarkably, for silvered nanoporous silica microparticles, the handedness conversion occurs at longer visible wavelengths, only after adsorption of molecules on the silver. Finite element analysis (FEA) allows matching the circular polarization (CP) conversion to dominant quadrupolar contributions, determined by the specimen size and complex susceptibility. We hypothesize that the damping accompanying the adsorption of molecules on the nanostructured silver facilitates the CP conversion. These results offer new perspectives in molecule sensing and materials tunability for light polarization conversion and control of light spin angular momentum at submicroscopic scale.

Plasmon polaritons, usually referred to as plasmons, are optically driven, collective oscillations of conduction electrons and are in most cases studied and exploited in metallic silver and gold nanoparticles, nanostructures, and interfaces.^1^ Plasmons can be accompanied by a substantial enhancement of the local electric field leading to surface-enhanced Raman scattering^2^ and fluorescence.^3^ Plasmons can also undergo spectral shift and broadening upon molecule adsorption and reconfiguration by chemical and refractive index damping.^2,3^ Damping of plasmons leads to a series of applications such as molecule sensors,^2,4−6^ hot-electron transfer-induced photocatalysis,^7^ photovoltaics,^8^ and can facilitate subwavelength confinement and propagation in microstructures in photonics.^9,10^ Apart from that, plasmonic materials are also designed and fabricated as metamaterials to control or convert the polarization of light, for example, by rotating the plane of polarization of linearly polarized light or by changing ellipticity and handedness.^11−13^

Propagating light also carries spin angular momentum with the two spin states of photon associated with right-hand and left-hand circular polarizations, as well as orbital angular momentum, associated with the transverse spatial coordinates. The total angular momentum is conserved and can be exchanged between spin and orbital angular momenta (i.e., spin–orbit interaction) and vice versa in nonparaxial conditions upon tight focusing and scattering by small particles or apertures.^14,15^ The spin–orbit interactions of light are rooted in Maxwell’s equations and are particularly relevant at subwavelength scales. Spin–orbit interactions justify, for example, the generation of optical vortices in liquid crystal droplets,^16^ the induction of orbital motion of nanoparticles with circularly polarized Gaussian beams,^17^ and the nonuniform polarization distribution of the light scattered by nanoparticles.^18^

Here, we report that a circularly polarized visible light focused with a low numerical aperture (NA) objective on plasmonic nanoparticles (e.g., 300 nm gold) dominantly scatters with opposite handedness in dark-field (DF). By solving the Helmholtz equation for the electric field using FEA and by also computing its propagation into the far-field, we correlate the CP conversion to dominant quadrupolar components, determined by the specimen size and complex susceptibility. In Ag-modified nanoporous isotropic silica (n-SiO_2_@Ag), the CP handedness at longer visible wavelength switch only upon the adsorption of 1-dodecanethiol (DDT) or ethynylaniline (EA). We propose that the damping accompanying the adsorption of molecule justifies the switch. These modified nanoporous particles are thus a template for high signal-to-noise ratio plasmonic polarimetry molecular sensors that are orientation independent and can tolerate a large level of spectral heterogeneity in their population. Furthermore, such nanostructured plasmonic materials have a large controllability that is also of interest in the development of new polarization conversion metamaterials^11,12,19,20^ and applications with light spin angular momentum.^14−18^

## Finite Element Analysis

**Figure 1:**
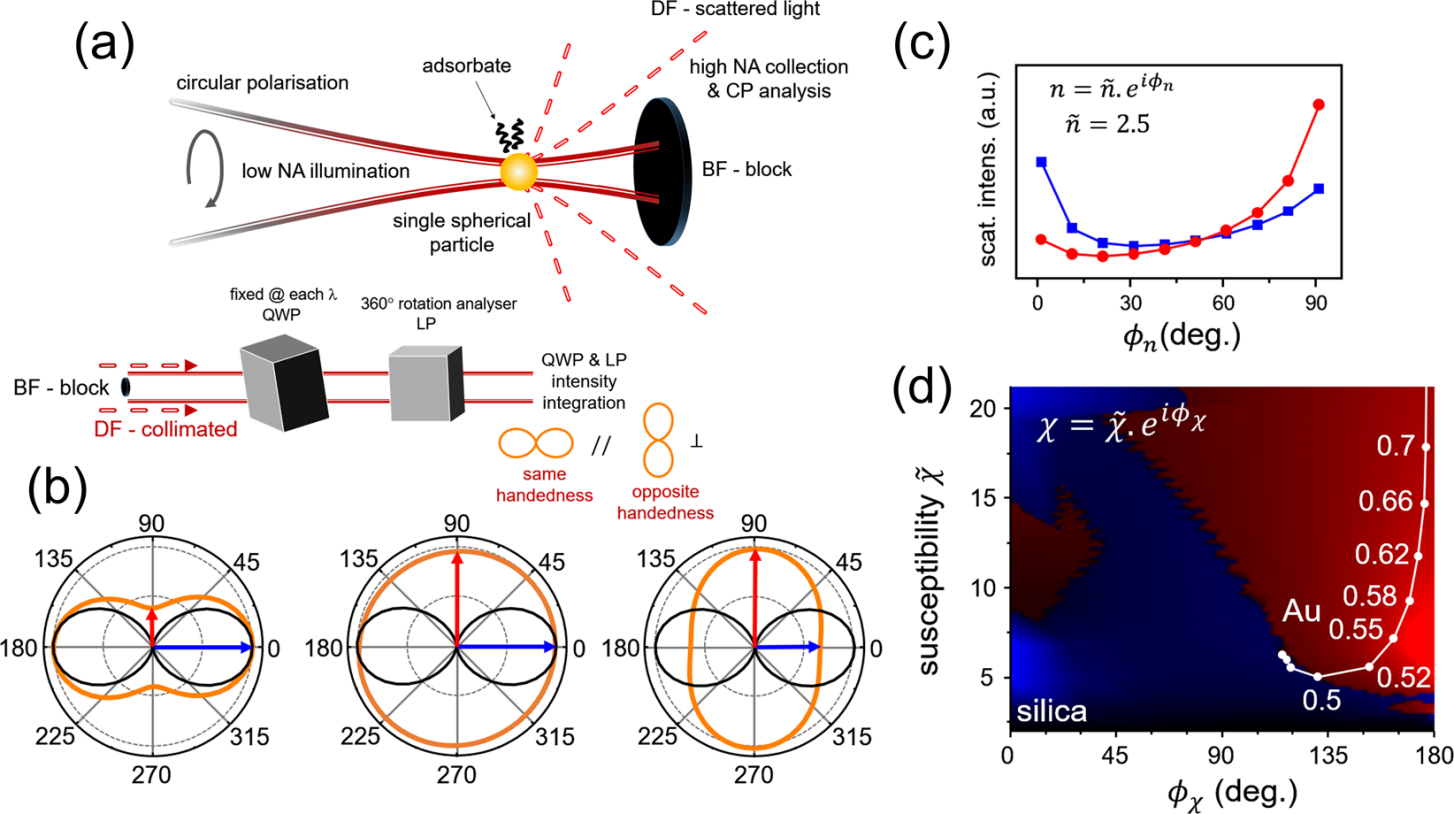
(a) A single particle is illuminated with a low NA focused Gaussian laser beam, and the scattered light is collected in the dark-field, with the bright-field blocked. For a circularly polarized input, the QWP is adjusted so that when the collimated scattered CP has the same or opposite handedness the polar plot with QWP-LP is horizontal (//) or vertical (⊥), respectively. (b) FEA-computed polar plots for circularly polarized light (wavelength 700 nm) scattered by a spherical particle (diameter 300 nm) characterized by a complex refractive index *n* = *ñ* *e*^*iϕ*_*n*_^ (*ñ* = 2.5). Black and orange lines are for low-angle BF collection and high-angle DF collection, respectively. ϕ_*n*_ = 0, 60, and 90° (from left to right). The blue and red arrows added onto the DF polar plots mark the // and ⊥ contributions. (c) Computed DF scattering intensity for the same particle as in (b) for // (blue) and ⊥ (red) collection. (d) Computed DF scattering intensity for the same particle as in (b,c), for an extended range of refractive index and with the axis marking the susceptibility χ = χ̃ *e*^*iϕ*_χ_^ instead. The regions where ⊥ (//) scattering dominates over // (⊥) are shown in red (blue). The susceptibility values of Au in the visible are marked with their labels expressed in micrometers.

A polarimetric scheme was implemented with an inverted DF transmission scanning microscope by focusing a circularly polarized Gaussian laser with a low NA objective, collecting the scattered light at high NA and directing it through a quarter-wave plate (QWP) for analysis with a rotating linear polarizer (LP) (Figure 1a). To facilitate the interpretation of the experimental results, we performed FEA using COMSOL Multiphysics. In all cases, for convenience, we aligned the QWP birefringent crystal and rotating LP in such a way that, when scattered and incident lights are of same handedness, the polar plot exhibits its main axis at 0° (//) and that, when they are of opposite handedness, the polar plot exhibits its main axis at 90° (⊥). In the manuscript, we can thus define that CP conversion is established when the ⊥ component dominates the // one.

**Figure 2:**
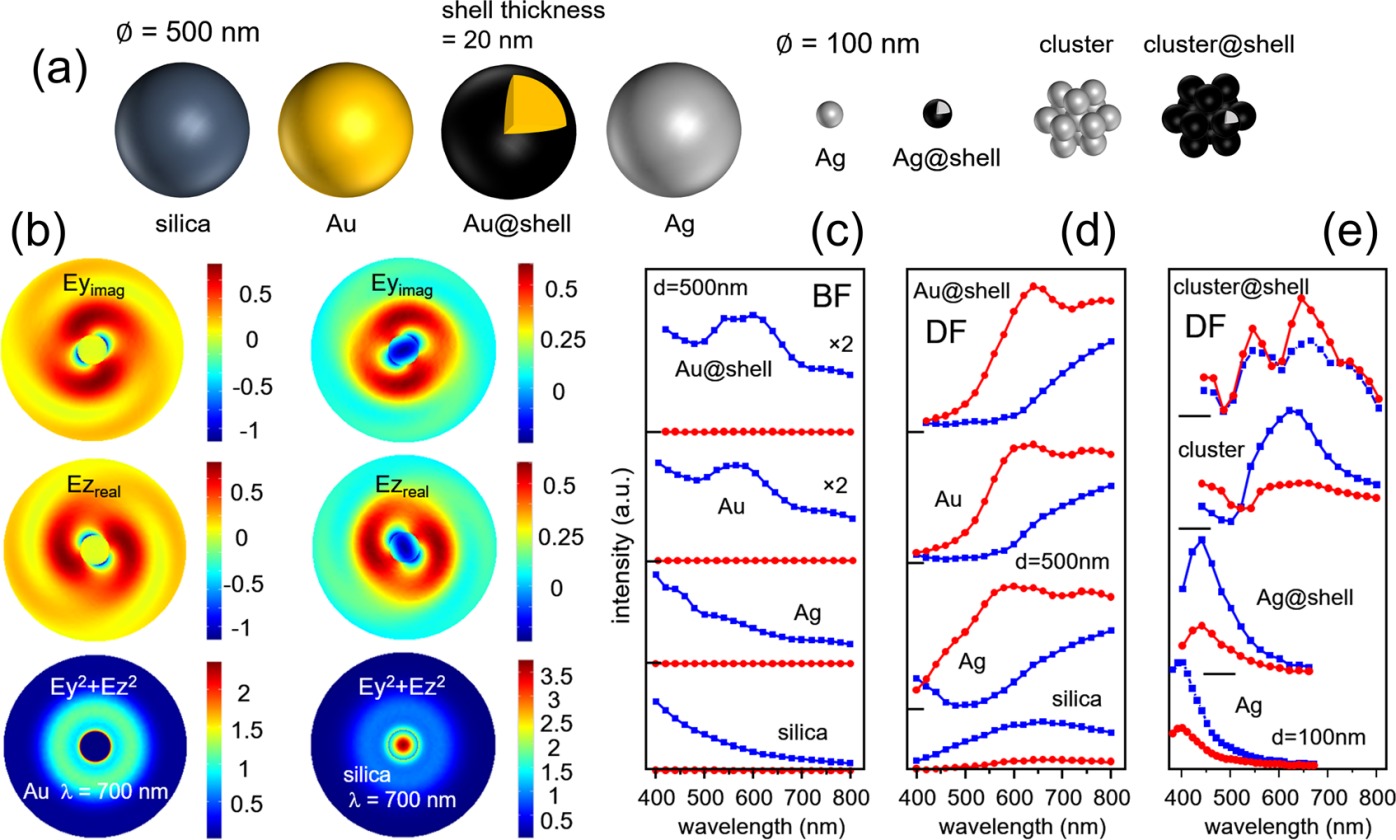
(a) Nanoparticles and materials analyzed by FEA. (b) Computed scattered fields and intensity in a transverse (*y,z*) plane (100 nm offset versus the particle center) for a 300 nm particle of Au with a wavelength of 700 nm and for a circularly polarized Gaussian background (left column). Same for silica (right column). (c) Computed spectra of the far-field scattered intensity with BF collection for 500 nm particles of silica, Ag, Au, and Au@shell. // and ⊥ CP components in blue and red, respectively. The intensities are plotted with a same arbitrary scale, magnified by ×2 for Au and Au@shell. The intensity zeros are marked by horizontal ticks. (d) Same as (c) for DF collection. (e) Same as (d) for a single 100 nm Ag nanoparticle and for a cluster of 13 Ag nanoparticles with and without shell.

FEA calculations allow us to investigate the relationship between polarimetric DF analysis and complex electric polarization in the material. The methodology for these calculations is detailed in the Supporting Information (SI) Section S1 (Figures S1 and S2). We consider the general case of scattering from a homogeneous spherical particle with a background circularly polarized Gaussian field. The sphere is defined with a complex-valued refractive index *n* = *ñ* *e*^*iϕ*_*n*_^ (or susceptibility χ = *n*^2^ – 1 = χ̃ *e*^*iϕ*_χ_^). We show in Figure 1b polar plots of the far-field projected scattering intensities for different values of ϕ_*n*_ (*ñ* = 2.5) with bright-field (BF) (black) and DF (orange) collection for a sphere of 300 nm diameter. The blue and red arrows mark the // and ⊥ CP components. The same color scheme is used in Figure 1c to plot the scattering intensities as a function of ϕ_*n*_ and also throughout this manuscript. Other susceptibility values were computed and the resulting // and ⊥ CP intensities are summarized in the colored map with axis ϕ_χ_ and χ̃ in Figure 1d. The values are shown in blue when the // component is larger than the ⊥ one and, conversely, the values are shown in red when the ⊥ component is larger than the // one. The values of susceptibility for gold in the visible^21^ are marked by white dots, and silica with its real susceptibility (*n* = 1.35) will appear in the bottom-left corner. Overall, the FEA reveals in DF that the polarization of a circularly polarized light scattered by a spherical 300 nm particle is dominantly ⊥ (i.e., opposite handedness, red) where ϕ_χ_ (or ϕ_*n*_) is larger. Moreover, which handedness dominates also depends on χ̃ and on the sphere size (see SI Figure S3 for a larger, 500 nm sphere). Thus, a 300 nm gold nanoparticle with a complex refractive index that is largely imaginary in the visible scatters with ⊥ CP in DF. Silica with a real and small refractive index scatters with // CP in the DF. Note that from these FEA simulations, the CP conversion is only expected in DF and the CP remains // in BF for all the values tested here (see black polar plot in Figure 1b, Figure 2, and SI Figure S4 for angular dependence for silica and Au).

We now discuss far-field spectra that we computed for a series of dielectric and metallic particles represented in Figure 2a. The series includes silica and Au particles, as well as Ag particles, single and in cluster. To clarify the origin of the CP conversion, we start with the analysis of the scattered electric field in and around an Au particle (Figure 2b). Specifically, we show in the left column the components Ey_imag_ and Ez_real_ of the field with the incident CP defined as (*j*, 1), for a 500 nm Au nanoparticle and with the wavelength set at 700 nm (see SI Figure S5 for a 300 nm Au nanoparticle). The intensity associated with these fields is also shown (bottom row). In the right column, we show the same for a silica particle. For gold, the near-fields are largest at the surface of the particle, in the vacuum. These fields have a strong quadrupolar contribution with complementary values in Ey_imag_ and Ez_real_ at the surface that can indeed be associated with an opposite CP (−*j*, 1). For silica, the fields retain stronger contributions within the particle boundaries with matching values in Ey_imag_ and Ez_real_ that can be associated with an unchanged CP (*j*, 1). Propagation in the far-field is thus expected to favor // CP for silica and ⊥ CP for Au. We note that scattering from quadrupole modes involves orbital angular momenta of +2ℏ and with the ⊥ CP spin at −1ℏ, the total angular momentum is +1ℏ. This value matches with the total angular momentum for the incidence // CP (spin, + 1ℏ) Gaussian (orbital, 0ℏ). Thus, from the conservation of the angular momentum one can rationalize an inversion of CP from // to ⊥, where quadrupole contributions dominate the scattering.^14−18^

We have plotted the // (blue) and ⊥ (red) CP components of the far-field scattering intensities in the BF and DF and across the visible spectrum (Figure 2c,d) for both silica and Au. BF and DF contributions were obtained by integrating the far-field radiation patterns, respectively, over a narrow and a hollow cone around the propagation axis (see SI Section S1 and Figure S2). We also show data computed for an Ag particle (500 nm) and for an Au particle covered with a thin (20 nm) dielectric shell (Au@shell), the latter being discussed further in the next paragraph. Overall, the spectra reveal that the CP scattering remains dominantly // for silica for all the computed wavelengths, while for all the 500 nm plasmonic materials Au, Ag, and Au@shell it is the ⊥ component that dominates. For low collection angles, the BF scattering remains with // CP at all wavelengths, irrespective of the samples. The // and ⊥ CP far-field scattering (BF and DF) spectra for Au and SiO_2_ follow the dependence on the refractive index discussed from Figure 1d. These spectra can be further rationalized from the near-field analysis made from Figure 2b that led us to conclude that for refractive index values such as those of Ag and Au in the visible, the quadrupolar contributions are significant. These are associated with a CP of opposite handedness and preferably detectable for high collection angles indeed. We verified that when the FEA is computed with a linearly polarized background field instead, the scattered fields do not exhibit any polarization change. This is expected since both CP handedness are present in the linearly polarized field and these will be affected equally, canceling any effect (SI Figure S6).

For Au and Au@shell, the FEA shows a plasmon band redshift from about 560 (Au) to about 580 nm (Au@shell) in the BF spectra (Figure 2c). This is expected from earlier works.^22,23^ Unremarkably, the computed far-field // and ⊥ CP spectra do not show an effect of the shell on the scattered polarization. However, we discuss now that a cluster of nanoparticles can exhibit a spectrally richer CP scattering with also a tunability of the CP conversion upon addition of dielectric shells. FEA // and ⊥ DF intensities are shown in Figure 2e for a 100 nm single Ag nanoparticle, for the same nanoparticle with a shell of 20 nm (Ag@shell), for a cluster of 13 Ag nanoparticles (diameter 100 nm, interparticle gap 10 nm), and for the same cluster with the nanoparticles covered by a 20 nm shell (cluster@shell). The single 100 nm Ag nanoparticle exhibits DF spectra peaking around 400 and 440 nm for Ag and Ag@shell, respectively, consistent with a plasmon resonance redshift in the presence of a thin film coating.^24,25^ With respect to the polarimetric analysis, the scattering of these Ag nanoparticles remains dominantly with // CP. For the cluster, the CP scattering is dominantly ⊥ below 520 nm and // above. Remarkably, the addition of a thin dielectric shell on the nanoparticles leads a CP scattering that is dominantly ⊥ across the entire visible spectrum. Thus, with these clusters there is a broad range of wavelengths (i.e., longer visible wavelengths) where one expects the polarimetric scheme to be very sensitive to surface modification.

## Dark-Field CP Polarimetry Experiments

**Figure 3:**
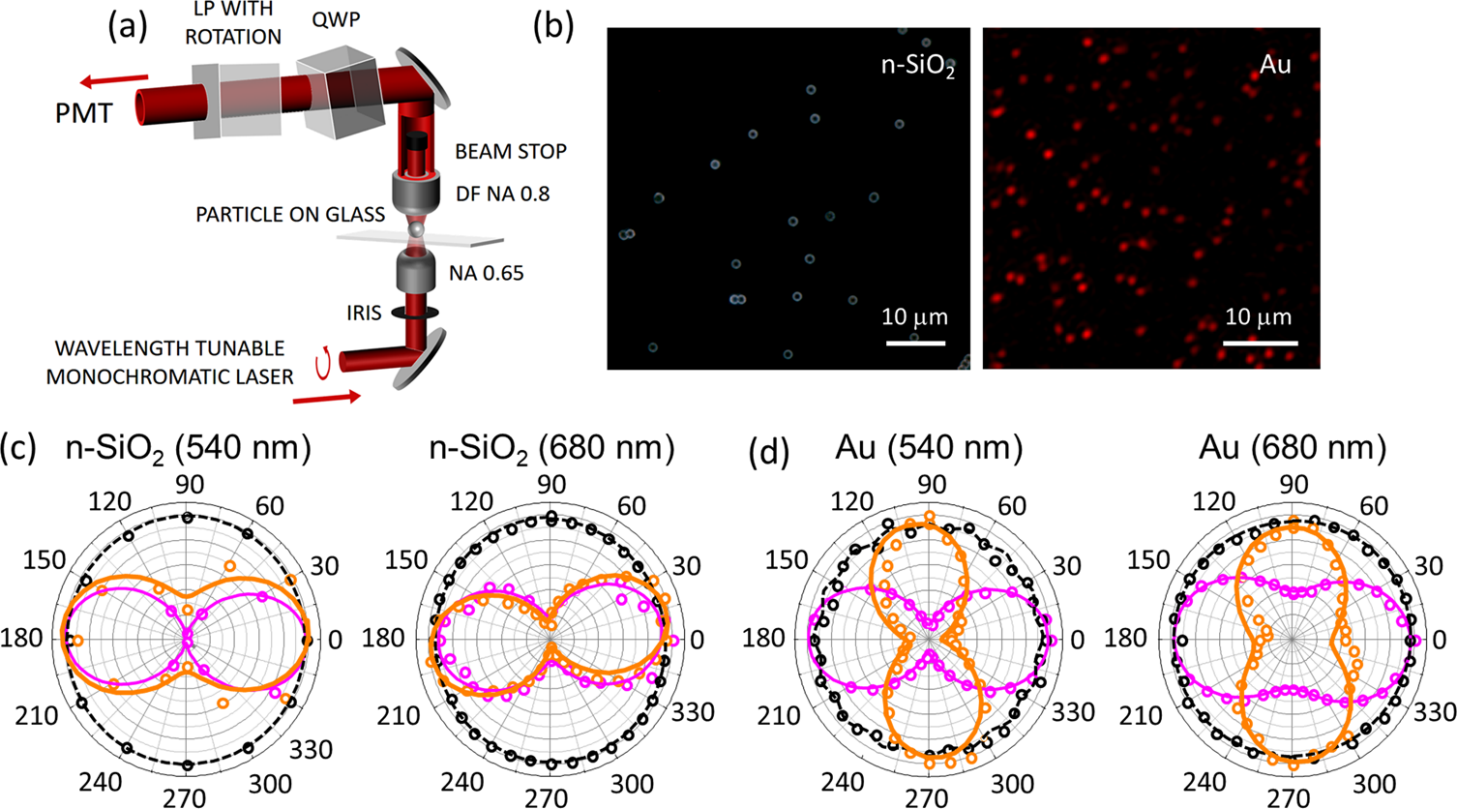
(a) Diagram of the DF transmission microscope with controllable input iris, removable BF beam stopper, wavelength tunable QWP, and LP analyzer on 360° rotation mount. (b) DF microscopy images of n-SiO_2_ microparticles (reflection white light using a Zeiss Axiovision equipped with a 20× Zeiss Epiplan 0.4 NA objective) and Au nanoparticles (300 nm) (with the setup shown in panel a). n-SiO_2_ and Au nanoparticles are readily dispersed on glass substrates. (c) Normalized single-particle polar plots (with QWP-LP) at 540 and 680 nm for n-SiO_2_ (BF in magenta, DF in orange, and depolarized DF background in black). (d) Same for Au nanoparticles.

The FEA conclusions are well reproduced in our experiments. The DF polarimetry experimental setup is presented in Figure 3a and further described in Methods (SI Section S2). We note that a particularity of our experiment is the low NA input and the high NA DF collection. First, we present the measurements for nanoporous silica microparticles (n-SiO_2_, diameter of about 1.5 μm, 92 Å average pore diameter, Glantreo) and Au nanoparticles (300 nm diameter, Sigma-Aldrich) dispersed on glass slides by drop casting (5 × 10^8^ particles/ml in citrate buffer) and drying (Figure 3b). We show in Figure 3c,d examples of normalized polar plots recorded on single silica microparticles and Au nanoparticles with the laser tuned at 540 and 680 nm. The polarization independent background (see black) measured away from particles was systematically removed from the data. This background is expected from random scattering in the glass substrate.

In the experiment, the BF data (see magenta) primarily measure the transmitted beam and not the scattered light. Indeed, BF images recorded on n-SiO_2_@Ag reveal that only about 20% of the incident power is lost when the focus is scanned across the microparticles (see SI Figure S7). As such, the observation of well-defined // polarizations in BF only highlights the quality of beam alignment, incident CP, and QWP alignment in the detection path and cannot be meaningfully compared with the FEA results discussed earlier (although experiments and FEA are seemingly in agreement). We also note that without the QWP, the BF polar plots are in good agreement with a circle confirming the CP (SI Figure S8). In DF (see orange in Figure 3) where the scattered light dominates and thus where a meaningful comparison with FEA can be drawn, the polar plots for n-SiO_2_ show the same polarization for all wavelengths and is used hereafter as reference for the CP // (for DF polarized images see SI Figure S9). Conversely, the DF polar plots for Au show a clear rotation with respect to n-SiO_2_, and now the ⊥ component dominates for all wavelengths (for DF images see SI Figure S10). For silica and Au particles, there is thus a remarkable experimental agreement with the FEA (Figure 2d).

**Figure 4:**
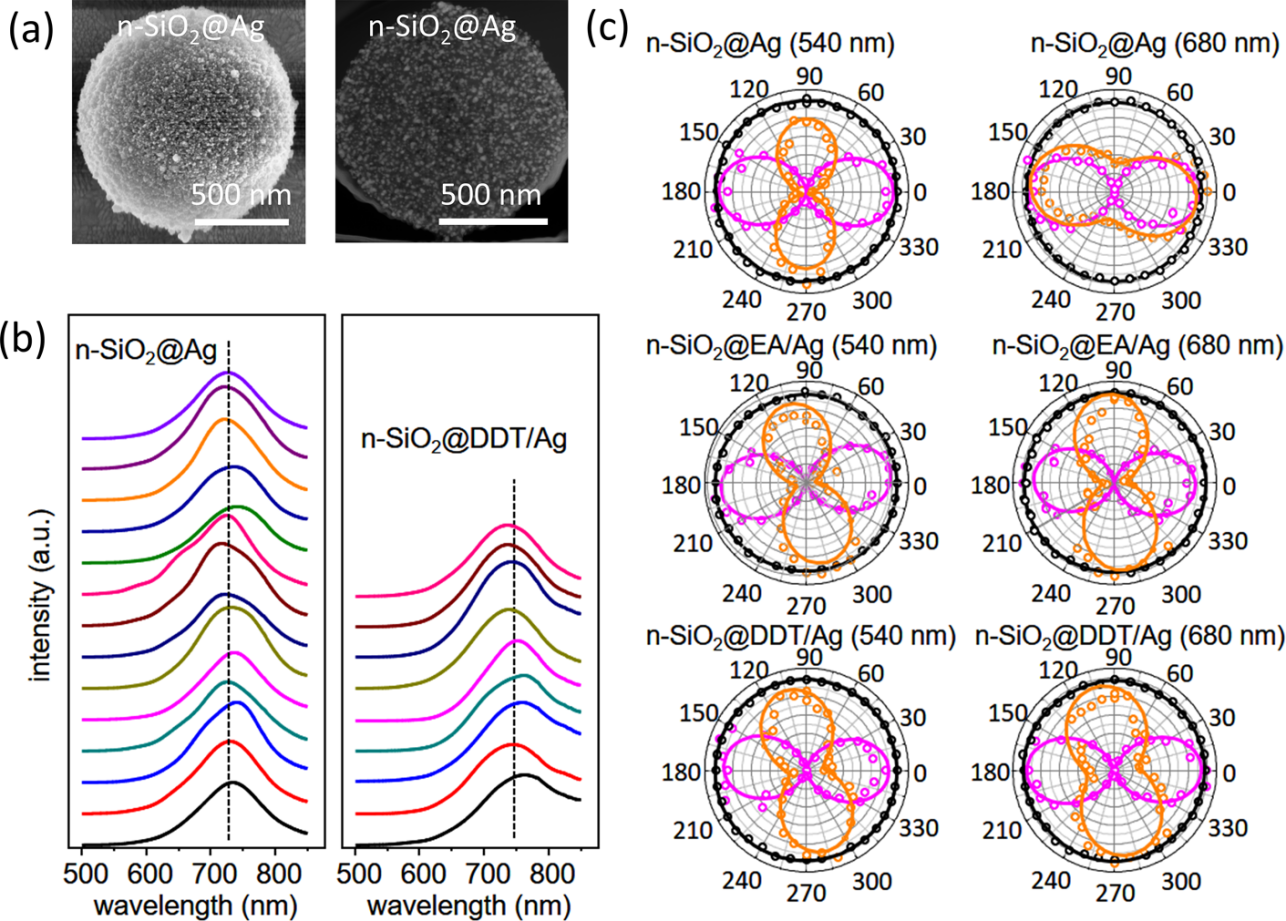
(a) SEM and HAADF-TEM of uncut and FIB-cut n-SiO_2_@Ag microsphere revealing Ag nanoparticle coverage at the sphere surface as well as in the pores. (b) Single particle scattering DF microspectroscopy of n-SiO_2_@Ag without and with DDT. The vertical dashed lines mark the average plasmon band central wavelength. (c) Normalized single particle polar plots for n-SiO_2_@Ag, n-SiO_2_@EA/Ag, and n-SiO_2_@DDT/Ag at 540 and 680 nm (BF in magenta, DF in orange, and depolarized DF background in black).

From FEA, one expects Ag nanoparticle clusters to exhibit a more wavelength-contrasted polarization response. In the experiment, we used Ag nanoparticles that are grown on the surface and in the pores of the n-SiO_2_ microspheres by an electroless reduction in ethanol with equimolar concentrations (1 mM) of AgNO_3_ and APTES.^26^ We established earlier by SERS that self-assembled monolayers of DDT, EA, and other molecules readily form on the embedded silver nanoparticles.^26^ A SEM image of a n-SiO_2_@Ag microsphere is shown in Figure 4a. It was recorded with a Helios G4 CX Dual Beam scanning electron microscope (SEM) equipped with focused ion beam (FIB) (Thermo Fisher Scientific). A FIB cross-section (gallium ion source) was prepared with the same instrument and imaged using a high-angle annular dark-field scanning transmission electron microscopy (HAADF-STEM). These images reveal a relatively uniform distribution of Ag nanoparticles at the surface and inside the nanoporous silica. No notable difference in Ag content or distribution was seen after the adsorption of DDT and EA. The n-SiO_2_@Ag microparticles disperse well on glass slides (Figure S11, SI) and low-angle collection broadband DF spectra (see SI Methods, Section S2) on single microspheres without and with DDT are presented in Figure 4b. The average plasmon wavelength redshift from about 729 to 747 nm with adsorption of DDT (n-SiO_2_@DDT/Ag). Also, the about 146 nm wide plasmon band in n-SiO_2_@Ag broadens to about 163 nm in n-SiO_2_@DDT/Ag. Plasmon band redshift and broadening suggest DDT-induced damping. However, the presence of multiple modes in the spectra precludes quantitative inference of the damping as done by other authors with smaller nanoparticles.^4−6,27^

The DF polar plots with incident CP for a n-SiO_2_@Ag particle in Figure 4c show that the scattered polarization is dominantly with ⊥ CP at 540 nm and // CP at 680 nm (for DF images and individual particles analysis see SI Figure S12; for additional plots on other single particles see SI Figure S13). The high-angle DF spectral dependence of both // and ⊥ components for a n-SiO_2_@Ag particle is reported in SI Figure S14a. The n-SiO_2_@Ag component dominates below about 580 nm, while the // component dominates above. The Ag clusters simulated by FEA are simpler than the experimental n-SiO_2_@Ag microparticles, since the hardware requirements for FEA scale up quickly with the high density and extended meshes required to simulate large systems of nanoparticles embedded in a porous dielectric matrix. However, the experimental observations are altogether in line with the FEA reported in Figure 2e; for shorter wavelengths, Ag clusters scatter dominantly with ⊥ CP, while they scatter with // CP at longer wavelengths. We also experimentally confirmed that no polarization conversion occurs with incident linear polarization (for spectra see SI Figure S14b, and for DF images see SI Figure S15), which is also in agreement with FEA.

After the adsorption of DDT or EA, the DF polar plots with CP are now ⊥ at both 540 and 680 nm, and the scattered CP at 680 nm has thus changed from // to ⊥. We note no significant difference between EA and DDT. It is remarkable that the microparticles heterogeneity does not affect the detection of the CP conversion, suggesting that the Ag nanostructuring is sufficiently homogeneous in the particles (for DF images and other particles see SI Figures S12, S15, S16 and S17). We verified that a smaller NA scheme, based on a NA 0.2 illumination and a NA 0.45 DF collection, also showed the same CP inversion upon addition of EA (see SI, Figure S16). This suggests that DF collection angles above about 25° are also suitable. From the FEA in Figure 2e, we inferred that a switch from dominant // CP to dominant ⊥ CP is reproduced by increasing the refractive index within a 20 nm thick shell around the Ag nanoparticles forming a cluster. Those FEA results clearly highlight the sensitivity to perturbation of Ag nanoparticle clusters. However, since DDT and EA molecular films are extremely thin (about 1–2 nm^28−30^), the perturbation in the experiment leading to the CP conversion when the molecules are added is not necessarily the same. Indeed, Figure 1d also suggests a dependence on the complex phase of the susceptibility. Moreover, Figure 2b clarifies that to induce CP conversion these changes in the susceptibility must be altering the relative contribution to the scattering of modes with different angular momentum. It is thus suggested that the damping^31^ accompanying the adsorption of molecules on the nanostructured silver justifies the CP conversion. This proposition is supported by the recent observation made by others that dipolar plasmon resonances exhibit higher damping than quadrupole ones upon adsorption of thiols.^32^ In the CP scattering model developed here, a sufficient relative increase in quadrupolar contributions will indeed achieve the CP inversion.

In this paper, we have demonstrated experimentally and justified by FEA that polarimetric (high-angle collection) DF far-field microscopy with // CP illumination shows significant conversion into ⊥ CP from the scattering by metal nanoparticles, such as 300 nm spherical gold particles. For nanostructured materials (n-SiO_2_@Ag), the surface modification accompanying molecular adsorption systematically induces the transition from // CP into ⊥ at longer wavelengths despite the spectral heterogeneity and complexity. Noteworthy, the switch in CP seen in the scattering is an example of spin to orbital angular momentum exchange mediated here by the plasmonic particles. The isotropic n-SiO_2_@Ag microparticles are relatively simple to prepare^26^ and with their high surface-to-volume ratio in Ag nanoparticles are promising microscopic platforms that are orientation-independent with respect to optical interrogation. Although a like-for-like benchmarking with other molecular sensor approaches will be required, the reduced sensor dimensions (i.e., in comparison to the surface plasmon resonance sensors in Kretschmann configuration^33^) can benefit in applications where smaller sample volumes are available and the isotropy suggests a potential use in microfluidic in-flow measurements. The adsorption of molecules at plasmonic materials is also a valid path to modify the macroscopic polarization of light across metamaterials and of other designed structures, with the outcome also determined here by the nanostructuring. From the FEA, we can infer that to exhibit controllable CP inversion, the plasmonic scatterer has to allow controllable scaling of the quadrupolar scattering. We showed here that relevant parameters are (when considering spherical particles) the size, the arrangement in clusters, and the dielectric environment. It is likely that other geometries favoring quadrupolar excitation such as nanocubes^34^ or nanotriangles^35^ are also suitable. As such, we envisage that the DF polarimetry will further be useful to investigate other microfabricated materials for a range of applications in plasmonics^19,20^ and light spin conversion.^14−18^

## Supporting Information Available

Far-field scattering polarimetry modeling (Section S1, Figures S1 and S2), description of experimental setup for laser DF polarimetry and broadband DF microspectroscopy (Section S2), additional simulation data (Figures S3–S6), BF image (Figure S7), additional experimental data (Figures S8, S13, and S14), and DF images (Figures S9–S12 and S15–S18) (PDF)
