# Supplementary material for: Circular Polarization Conversion in Single Plasmonic Spherical Particles
Source: Nano Lett. 2022 Feb 3;22(4):1504–10. doi: 10.1021/acs.nanolett.1c03848 (PMC8880373; doi:10.1021/acs.nanolett.1c03848)
Supplement: Supplementary file 1 — nl1c03848_si_001.pdf [file nl1c03848_si_001.pdf]

Supplementary Materials

Circular polarization conversion in single plasmonic spherical particles

Pritam Khan[1], Grace Brennan[1], Zhe Li[1,3], Luluh Al Hassan[2], Daragh Rice[1], Matthew Gleeson[1], Aladin A. Mani[1], Syed A.M. Tofail[1], Hongxing Xu[3], Ning Liu[1,*] and Christophe Silien[1,*]

[1]*Department of Physics and Bernal Institute, University of Limerick, Limerick, V94 T9PX, Ireland*

[2]*Department of Chemical Sciences and Bernal Institute, University of Limerick, Limerick, V94 T9PX, Ireland*

[3]*School of Physics and Technology, Institute for Advanced Studies and Center for Nanoscience and Nanotechnology, Wuhan University, Wuhan, 430072, China*

*Corresponding Authors: Christophe Silien, e-mail: christophe.silien@ul.ie

Ning Liu, e-mail: ning.liu@ul.ie

S1. Far-field scattering polarimetry modelling

We computed the scattered field $\boldsymbol{E_{scat}}$ from spherical objects and clusters of spherical objects, irradiated with a background field $\boldsymbol{E_b}$, using finite the element analysis (FEA) software COMSOL Multiphysics®'s electromagnetic module, in the frequency domain, solving the time-independent Helmholtz equation at fixed wavelength λ:

$$\boldsymbol{\nabla} \times (\boldsymbol{\nabla} \times \boldsymbol{E}) - k^2 \varepsilon_r \boldsymbol{E} = 0 \quad \textbf{(eq.1)}$$

where $\varepsilon_r = (n - i\kappa)^2$, $k = n\frac{2\pi}{\lambda}$ and $\boldsymbol{E} = \boldsymbol{E_b} + \boldsymbol{E_{scat}}$. The background field was set as Gaussian aligned and centered onto the spherical objects:

$$\boldsymbol{E_b}(x, y, z) = \boldsymbol{E_{b0}} \frac{w_0}{w(x)} exp\left[\frac{y^2+z^2}{w^2(x)}\right] exp\left[-jkx - jk\frac{y^2+z^2}{2R(x)} + j\eta(x)\right] \quad \textbf{(eq.2)}$$

where $w(x) = w_0\sqrt{1 + \left(\frac{x}{x_0}\right)^2}$, $R(x) = x\left[1 + \left(\frac{x_0}{x-x_0}\right)^2\right]$, $\eta(x) = tan^{-1}\left(\frac{x}{x_0}\right)$, and $x_0 = \frac{k_0 w_0^2}{2}$. The vector field amplitude was respectively $\boldsymbol{E_{b0}} = (0, j, 1)$ or $\boldsymbol{E_{b0}} = (0,1,1)$, for circular or linear polarisation, and the waist was $w_0 = \frac{\lambda}{2\times 0.4}$ to account for the low NA objective used to focus the monochromatic laser beam on the sample in the experiment.

The computation domain was defined as a cylinder aligned with the direction ($\vec{x}$) of propagation of $\boldsymbol{E_b}$ (Figure S1), and delimited by scattering boundary condition surfaces all around, to prevent reflection of the scattered field. Background Gaussian and particle/cluster under study were both centered on the coordinate origins, also at the center of the computation cylindrical domain. The mesh cells maximum size was kept less than $\lambda/6$ and minimum allowed down to $\lambda/100$.

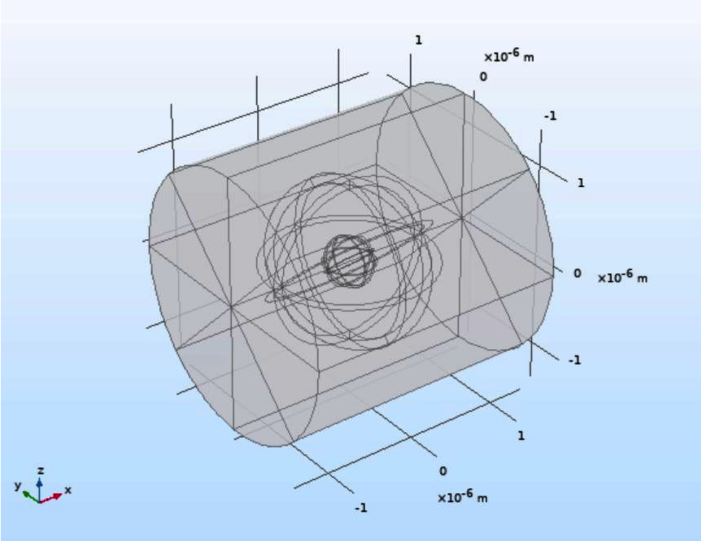

Figure S1. COMSOL computation domain, including a spherical object (500 nm diameter with concentric 20 nm shell) in the center and a 1.0 µm sphere defining the surface S for far-field computation. The cylindrical domain was of radius 1.4 µm and length 2.8 µm.

The far-field $\boldsymbol{E_{far}}$ (Figure S2, left panel) was computed using COMSOL's native functionality based on Stratton-Chu formulation:

$$\boldsymbol{E_{far}}(\theta,\phi) = \frac{jk}{4\pi}\boldsymbol{r_0} \times \int \left[\boldsymbol{n} \times \boldsymbol{E_S} - \sqrt{\frac{1}{\varepsilon}}\,\boldsymbol{r_0} \times (\boldsymbol{n} \times \boldsymbol{H_S})\right] exp(\,jk\,\boldsymbol{r}.\boldsymbol{r_0})\, dS \qquad \textbf{(eq.3)}$$

Here, the computed far-field only accounts for $\boldsymbol{E_{scat}}$. The electric and magnetic fields $\boldsymbol{E_S}$ and $\boldsymbol{H_S}$ are thus the values of the scattered fields on the far-field computation surface S. $\boldsymbol{r_0}(\theta,\phi)$ is a unit vector pointing from the origin (center of far-field integration sphere) marking the direction of $\boldsymbol{E_{far}}$, $\boldsymbol{n}$ is the unit vector normal to the surface S, and $\boldsymbol{r}$ is the radius vector of the surface S.

The complex-valued far-field $\boldsymbol{E_{far}}(\theta,\phi)$ were exported into Matlab for polarimetric analysis. Specifically, we computed the polarisation components using Jones matrices. In line with the experiment, the quarter

waveplate (QWP) axis was oriented so that the circular polarised background field $(0, j, 1)$ is made linear polarised $(0,1,1)$, and the QWP was followed by a linear polariser (LP) with alignment defined by the angle θ_{LP}, zeroed onto the $(0,1,1)$ axis, and only affecting the in-plane, $\vec{y}$ and $\vec{z}$, components of the field. The resulting $\boldsymbol{E_{QWP}}$ field writes:

$$\begin{pmatrix} \boldsymbol{E_{QWP}.y} \\ \boldsymbol{E_{QWP}.z} \end{pmatrix} = \begin{pmatrix} sin^2(\theta_{LP}) & sin(\theta_{LP})\, cos(\theta_{LP}) \\ sin(\theta_{LP})\, cos(\theta_{LP}) & cos^2(\theta_{LP}) \end{pmatrix} \begin{pmatrix} -j & 0 \\ 0 & 1 \end{pmatrix} \begin{pmatrix} \boldsymbol{E_{far}.y} \\ \boldsymbol{E_{far}.z} \end{pmatrix}$$

$$= \begin{pmatrix} -j\, sin^2(\theta_{LP}) & sin(\theta_{LP})\, cos(\theta_{LP}) \\ -j\, sin(\theta_{LP})\, cos(\theta_{LP}) & cos^2(\theta_{LP}) \end{pmatrix} \begin{pmatrix} \boldsymbol{E_{far}.y} \\ \boldsymbol{E_{far}.z} \end{pmatrix} \quad \textbf{(eq.4)}$$

The field $\boldsymbol{E_{LP}}$, in the absence of quarter waveplate writes:

$$\begin{pmatrix} \boldsymbol{E_{LP}.y} \\ \boldsymbol{E_{LP}.z} \end{pmatrix} = \begin{pmatrix} sin^2(\theta_{LP}) & sin(\theta_{LP})\, cos(\theta_{LP}) \\ sin(\theta_{LP})\, cos(\theta_{LP}) & cos^2(\theta_{LP}) \end{pmatrix} \begin{pmatrix} \boldsymbol{E_{far}.y} \\ \boldsymbol{E_{far}.z} \end{pmatrix} \quad \textbf{(eq.5)}$$

The signal at the detector is then written as proportional to the integral of the field squared norm computed over all (θ, ϕ) combinations forming the bright field (BF) or dark field (DF) angular apertures (Figure S2, right panel):

$$I_{QWP/LP} = \iint_{BF/DF} \left\| E_{QWP/LP} \right\|^2 sin(\theta)\, d\theta\, d\phi \quad \textbf{(eq.6)}$$

For BF, we integrated a cone of angular aperture 5° around the direction of propagation $\vec{x}$. For DF, we integrated over an annular aperture (between 70 and 75°) around the same direction, or as specified in the text.

The materials refractive index was defined as n = 1.35 (real) for silica and using in-built complex values for Ag and Au. Dielectric films were set to n = 1.3-1.7 (real). In the other cases, the refractive index has been defined as $n = \tilde{n}\, e^{j\phi_n}$ (see main text).

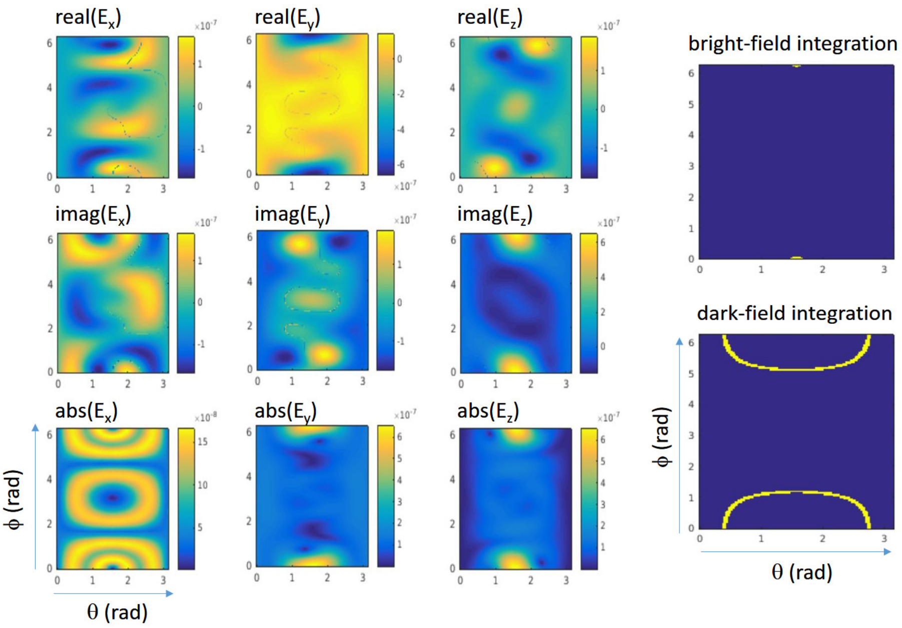

Figure S2: (left) Far-field electric field complex components in spherical coordinates for a 500 nm Au sphere and background field set at a wavelength of 540 nm. (right) Bright-field and dark-field integration spaces (yellow).

S2. METHODS

Laser, high-angle DF polarimetry

We set up a transmission optical microscope (Figure 3(a)) where a collimated monochromatic laser beam is focused with a long working distance low NA objective (50× Leitz PLAN L, NA = 0.60, further limited with an adjustable input iris). The scattered light is collected by a DF objective (50× Olympus UMPlanFl, NA = 0.8) with the BF collection core occulted with a removable beam stopper for DF measurements. The intensities were measured with a photomultiplier tube (PMT) (Hamamatsu, E717-500). The input iris was adjusted to maximize BF transmission whilst minimizing any stray light in DF when the focus is away from particles. The linear polarized incident laser light was made CP using a wavelength-tunable QWP before entering the microscope (not shown in the sketch) and a second QWP, adjusted for realizing opposite handedness was placed in the transmitted detection path before the PMT. The QWP in the detection path is paired with a 360° rotatable LP to produce the experimental polar plots of scattered intensity (QWP-LP). For each experiment and wavelength, the incident CP (Figure S7, SI) and QWP alignment were verified in BF with the beam focused away from any particles. This was done, first, by recording a uniform intensity for all LP angles in the absence of detection QWP and, second, by recording a linear polarized light in the presence of detection QWP, for all LP angles, with the dominant polarization axis aligned with that of the incident laser linear polarization (i.e., // or 0°). The sample was mounted on a piezo-scanner for focusing and scanning. To generate polar plots, we recorded a series of images with the detection LP set at a specific angle and integrated the intensity over individual particles in each angle frame. Thus, the polar plots are established for single particles. The tunable laser source available in this work was an optical parametric amplifier (OPA, 1 kHz, pulse duration ca. 3 ps) TOPAS from Light Conversion pumped by a Ti-Sapphire regenerative amplifier from Coherent. The power on the sample was kept at 0.1 μW.

Broadband, low-angle DF micro-spectroscopy

The transmission setup consisted of an Olympus 1.2-1.4 NA dark-field condenser with type-F immersion oil and a 0.9 NA 100× Olympus objective (MPLFLN100×) for collection. A 150 W halogen lamp (OSL2, Thorlabs) was used for illumination, with a Princeton instruments Acton series SP2300 spectrometer (600/mm grating blazed at 750 nm) directed onto a cooled PIXIS 256 CCD camera for detection. The grating was moved to collect the spectrum in 100 nm increments with 50 nm overlap. Each section was acquired for 10 ms and accumulated twice. The dark-field spectra were corrected by subtracting the spectrum of a nearby area (clean and free of particles) and dividing by the white light spectrum.

NA and polarization error

We have considered whether the high collection angle of the scattered light and its subsequent collimation affect the polarization that is measured. The issue stems from the mixing of longitudinal polarization into transversal polarizations at high angle. To evaluate the impact of this in our experiment, where the NA is 0.8, we have computed the depolarization of a beam of linear polarization when it is focused with a high NA lens using the Debye-Wolf integral [1]. The rationale is that the contribution mixing will be the same upon collection, and that the model allows us thus to assess the extent of non-ideality with the DF polarimetry. The depolarization is expressed as the ratio of intensity with longitudinal polarization to the intensity with transversal polarization when the beam incident on the lens is of linear polarization. We computed these numbers for a fully illuminated aperture and for an annular aperture (where only 20% of the full aperture is kept). The results are summarized in the table below and show that the non-ideality can be estimated as 15% for a NA of 0.8, that matches with the experiment. As such, we decided not to introduce any postprocessing correction in both experiments and simulations. We note however that the non-ideality/mixing with longitudinal polarization components increases rapidly when higher NAs are considered, and that one expected 62% of longitudinal contribution for a NA of 1.4. As such, should the

scheme be redeveloped with higher NAs, one would certainly have to account for the non-ideality with a suitable form of post-processing.

NA	annular (20%) I_{long} / I_{trans}	full aperture I_{long} / I_{trans}
**0.4**	0.015	0.005
**0.8**	0.14	0.065
**1.4**	0.62	0.29

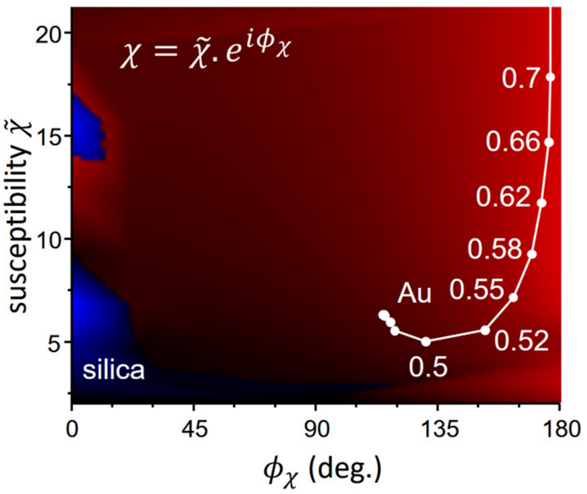

Figure S3: FEA-computed scattered intensity at high collection angles (DF) for a 500 nm (diameter) particle for an extended range of refractive index and with the axis marking the susceptibility $\chi = \tilde{\chi}e^{i\phi_\chi}$. The focus was defined with a wavelength of 700 nm. The regions where ⊥ (//) dominates over // (⊥) are shown in red (blue). The susceptibility values of Au in the visible are marked with their labels expressed in μm.

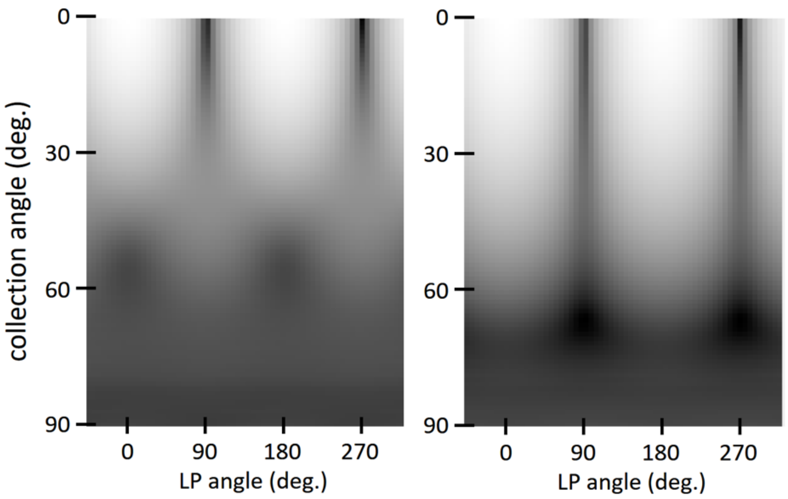

Figure S4: FEA-computed (wavelength 700 nm) far-field scattering intensities for a 500 nm Au (left) and silica (right) particle. The intensities are presented with a gray scale (larger intensities in white, log. scale), with the horizontal axis corresponding to the orientation angle of the LP analyzer (QWP in place), and with the vertical axis matching with the DF collection angle (10 degrees integration step and normalization by the captured angular area). Here, above ca. 45 degrees, Au exhibits a dominantly ⊥ scattering while silica remains //.

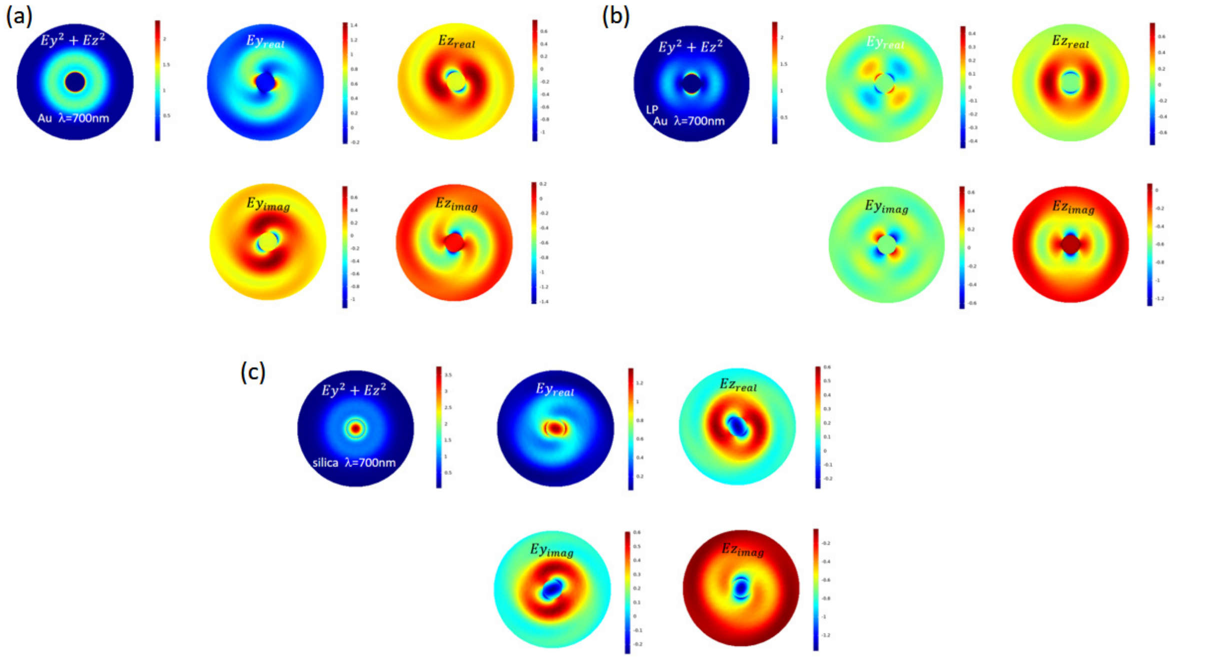

Figure S5: Electric field in a transverse (y, z) plane at an offset of 100 nm with respect to the particle center computed by FEA. The particle diameter is 300 nm and the wavelength 700 nm. (a) Au particle with CP $(j, 1)$ Gaussian background field. (b) Au particle with LP $(0,1)$ Gaussian background field. (c) Silica particle with CP $(j, 1)$ Gaussian background field.

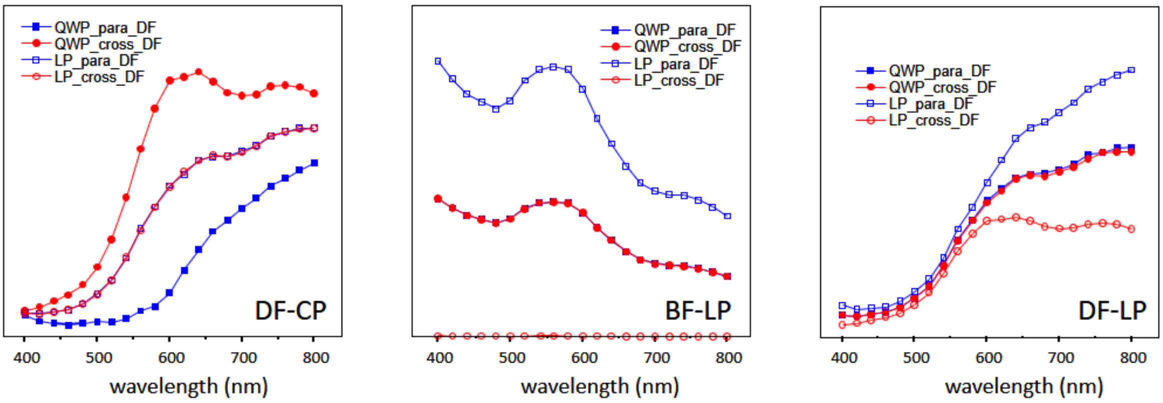

Figure S6: Polarimetric analysis of the FEA-computed far-field scattering with a 500 nm Au nanoparticle. Analysis with QWP-LP // and ⊥ components (closed blue squares and closed red circles, respectively) and analysis with LP only (i.e., without QWP in the detection) // and ⊥ components (open blue squares and open red circles, respectively). (a) CP illumination and dark-field collection. (b) Linear polarized illumination and bright-field collection. (c) Linear polarized illumination and dark-field collection.

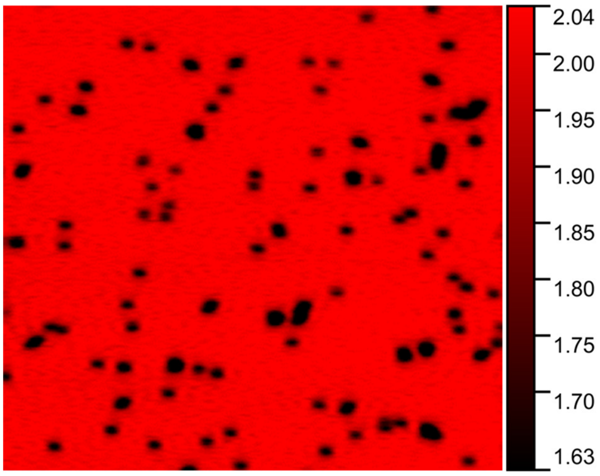

Figure S7. BF transmission image of n-SiO_2@Ag microspheres at 680 nm, recorded with the setup described in Figure 3a, with the BF block removed. The incident polarization is circular, and the intensity was recorded with the QWP-LP pair for parallel (//) component. Image horizontal size = 100 μm.

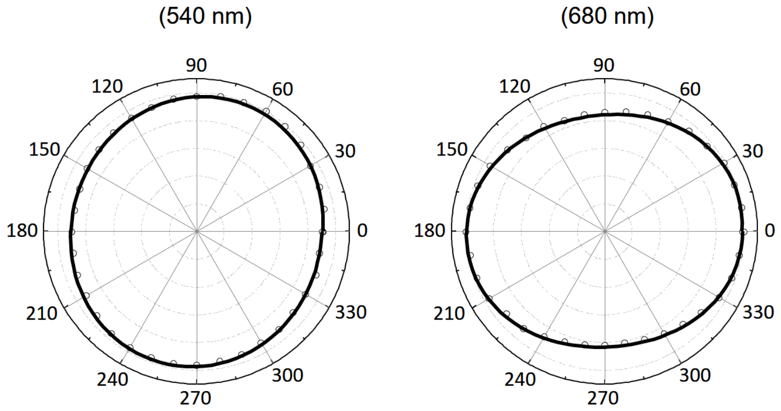

Figure S8. Experimental confirmation of CP alignment at 540 and 680 nm in BF, with the beam focus away from any particles with LP analyzer only (i.e., in the absence of detection QWP).

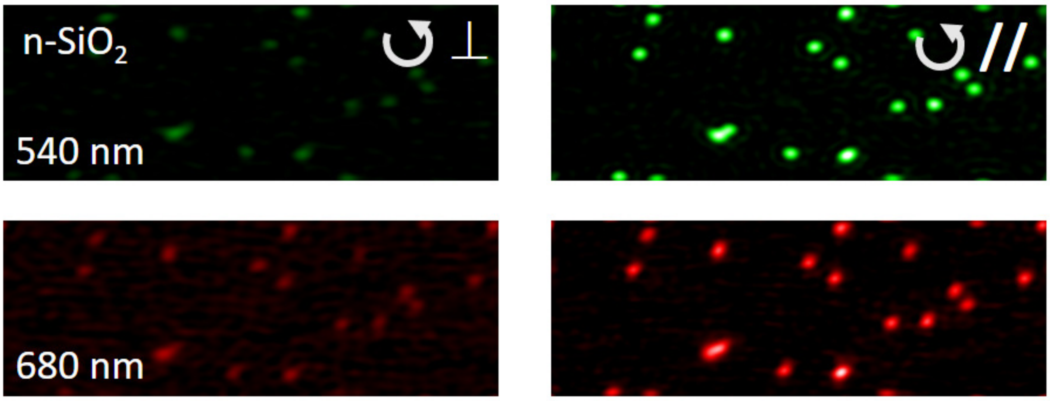

Figure S9. DF images of n-SiO_2 microspheres at 540 and 680 nm (from top to bottom). The incident polarization is circular, and the intensity recorded with the QWP-LP pair for perpendicular (⊥) and parallel (//) components (left and right column, respectively). For such n-SiO_2 microspheres, the // component dominates for all wavelengths (i.e., no remarkable change in polarization). All the images were recorded over a same location of the glass slide on which the microspheres have been dispersed. (All images horizontal size = 100 μm).

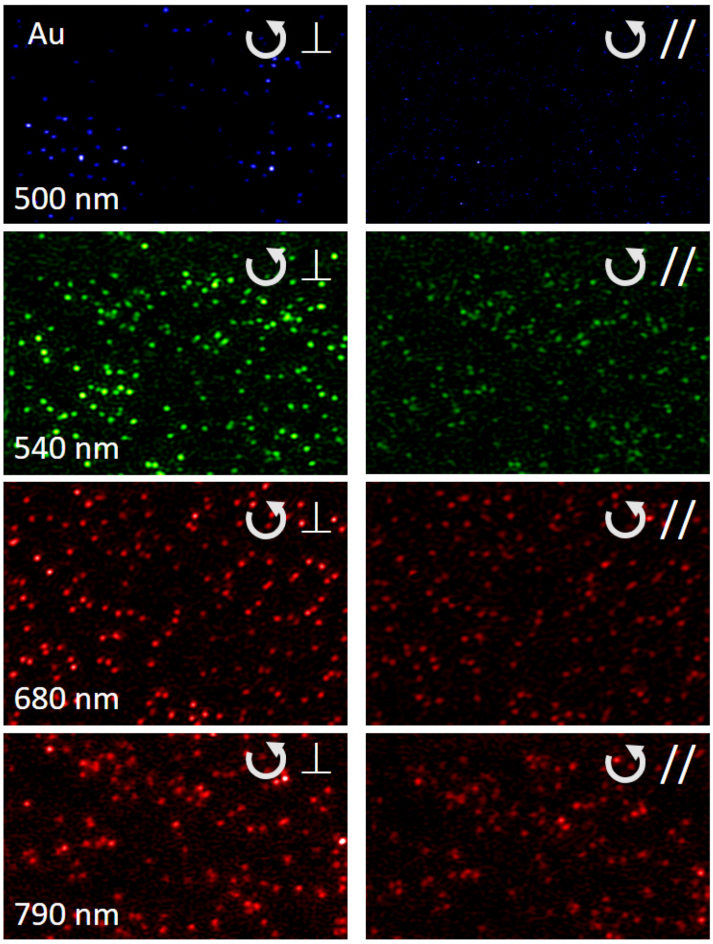

Figure S10. DF images of Au nanoparticles (diameter = 300 nm) at 500, 540, 680 and 790 nm (from top to bottom). The incident polarization is circular, and the intensity recorded with the QWP-LP pair for perpendicular (⊥) and parallel (//) components (left and right column, respectively). For such Au nanoparticles, the ⊥ component dominates for all wavelengths. All the images recorded at a particular wavelength were measured over the same location of the glass slide on which the nanoparticles have been dispersed. (All images horizontal size = 100 μm).

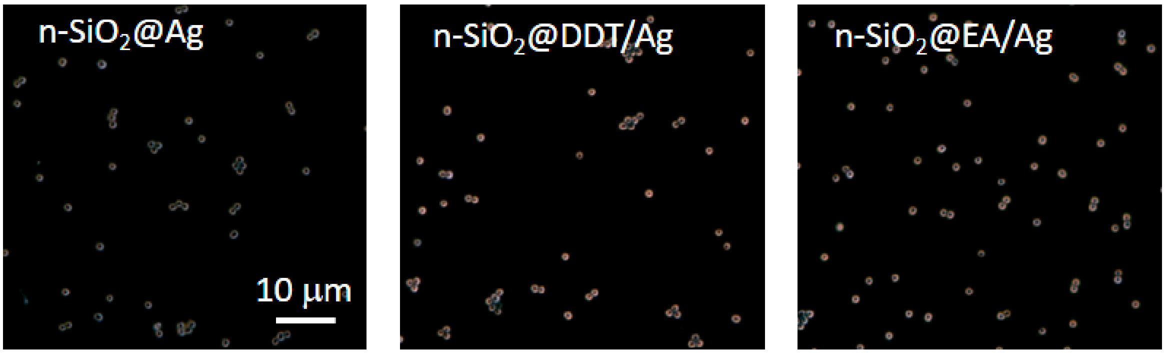

Figure S11. Broadband white light, wide-field, DF 0.8 NA reflection microscopy images showing good dispersion of the modified n-SiO_2@Ag microspheres on a glass slide after drop-casting and drying: without additional molecules (left), with DDT (center), and with EA (right).

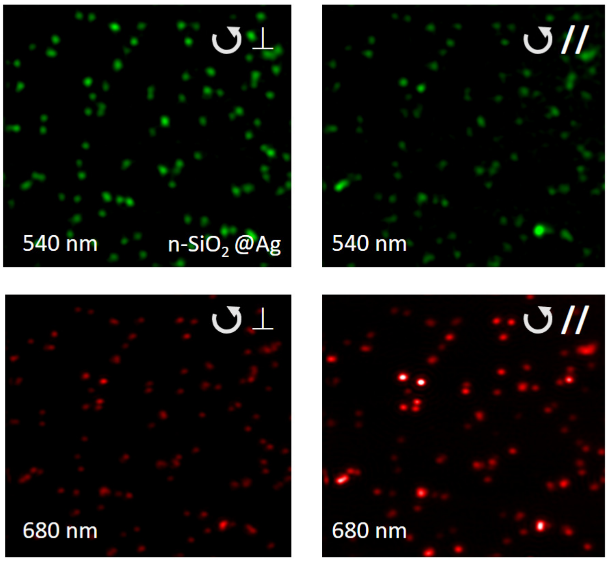

Figure S12. DF images of n-SiO_2@Ag microspheres at 540 and 680 nm (top and bottom row). The incident polarization is circular, and the intensity recorded with the QWP-LP pair for perpendicular (⊥) and parallel (//) components (left and right column, respectively). At 540 nm, the ⊥ component dominates, while at 680 nm the // component dominates. All images are recorded over the same location of the glass slide on which the microspheres have been dispersed. (All images horizontal size = 110 μm).

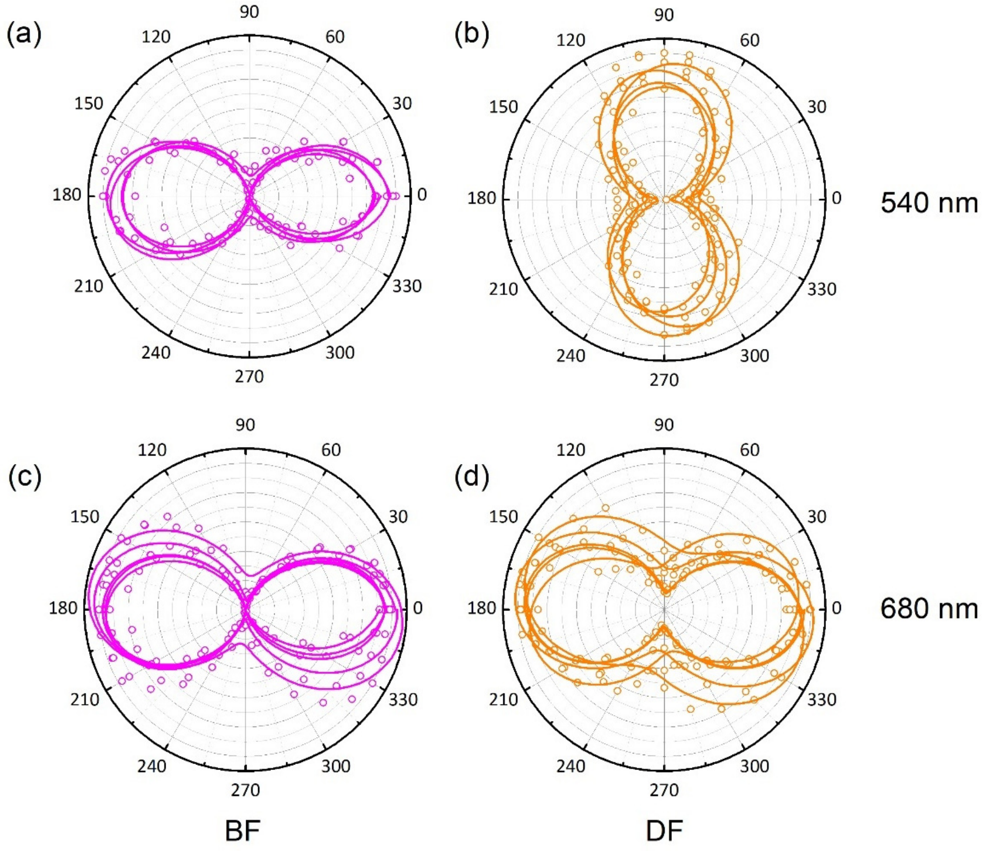

Figure S13. Series of (normalized) polar plots recorded for different n-SiO_2@Ag particles. (a) 540 nm BF in magenta (4 different particles). (b) 540 nm DF in orange. (c) BF 680 nm in magenta (5 different particles). (d) DF 680 nm in orange.

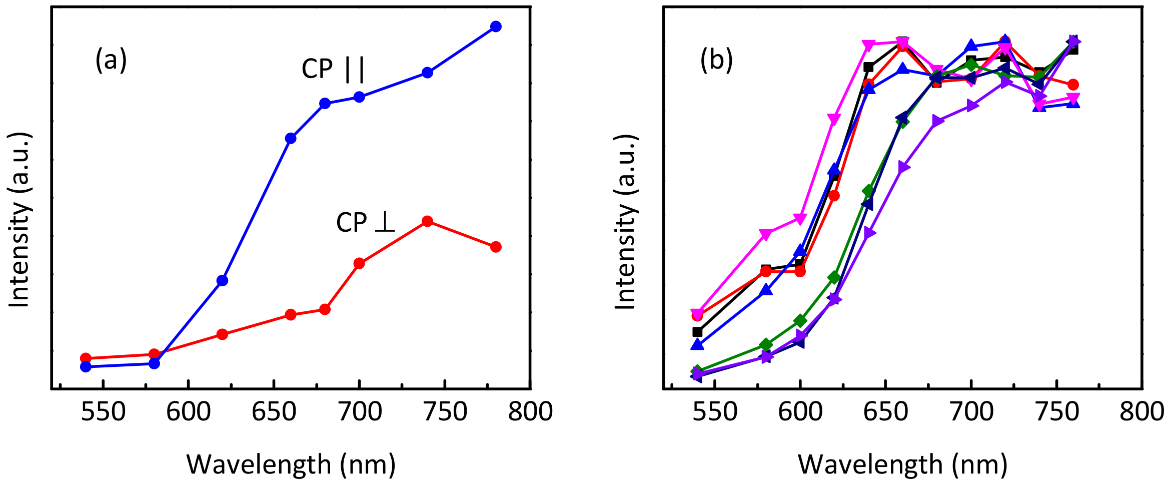

Figure S14. High-angle DF spectra for n-SiO_2@Ag microparticles. (a) // and ⊥ components with incident CP for single particle. (b) Total intensity with input linear polarization for several particles.

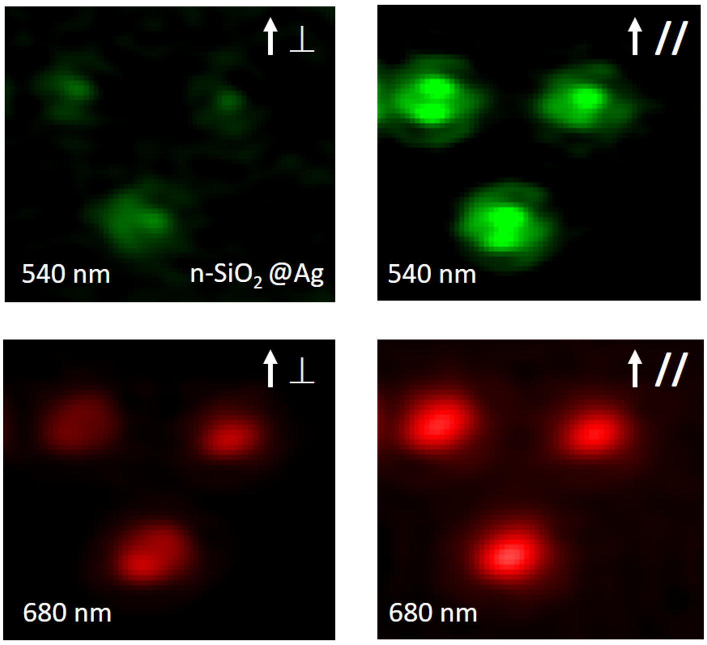

Figure S15. DF images of n-SiO_2@Ag microspheres at 540 and 680 nm (top and bottom row). The incident polarization is linear, and the intensity recorded with only the LP for perpendicular (⊥) and parallel (//) components (left and right column). In these conditions, the // component dominates for all wavelengths tested in this study. All images are recorded over the same location of the glass slide on which the microspheres have been dispersed. (All images horizontal size = 12.5 µm).

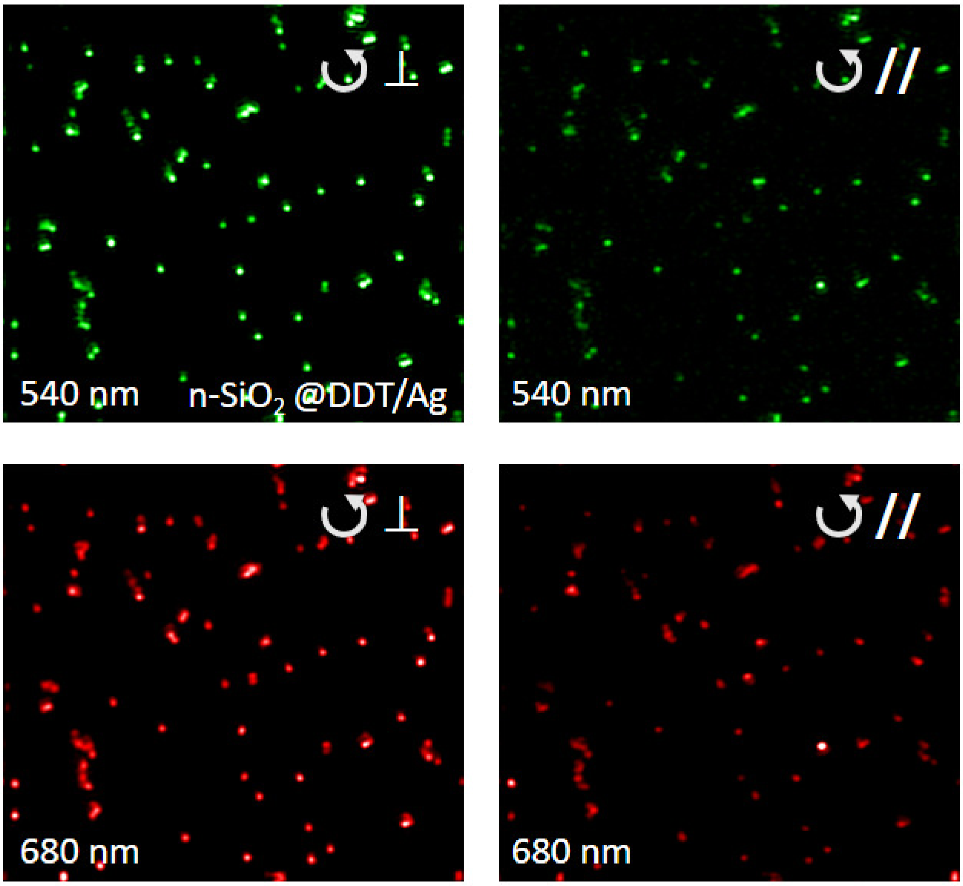

Figure S16. DF images of n-SiO_2@DDT/Ag microspheres at 540 and 680 nm (top and bottom row). The incident polarization is circular, and the intensity recorded with the QWP-LP pair for perpendicular (⊥) and parallel (//) components (left and right column, respectively). At both 540 and 680 nm, the ⊥ component dominates. All images are recorded over the same location of the glass slide on which the microspheres have been dispersed. (All images horizontal size = 110 μm).

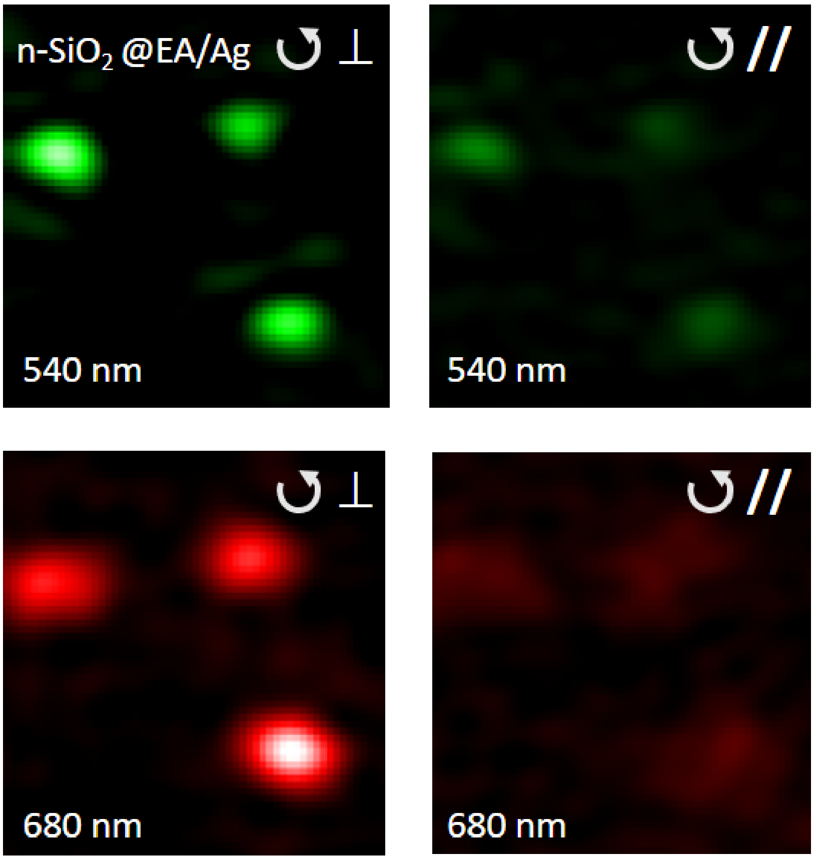

Figure S17. DF images of n-SiO_2@EA/Ag microspheres at 540 and 680 nm (top and bottom row). The incident polarization is circular, and the intensity recorded with the QWP-LP pair for perpendicular (⊥) and parallel (//) components (left and right column, respectively). At both 540 and 680 nm, the ⊥ component dominates. All images are recorded over the same location of the glass slide on which the microspheres have been dispersed. (All images horizontal size = 10 μm).

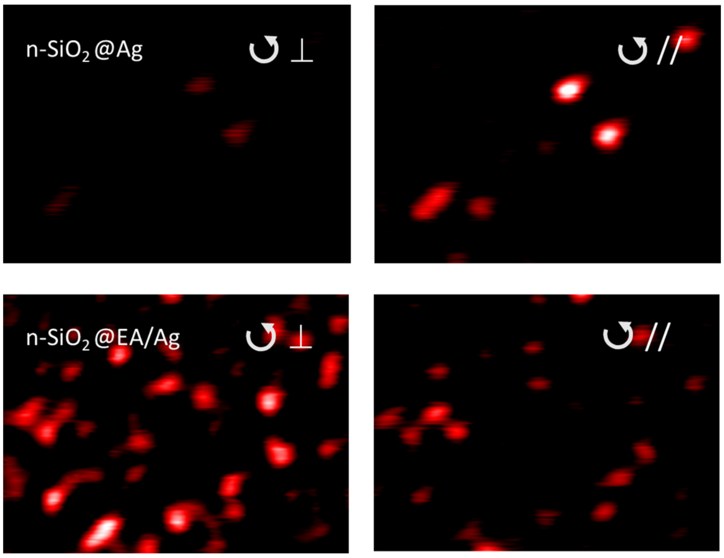

Figure S18. DF images of n-SiO_2@Ag and n-SiO_2@EA/Ag microspheres (top and bottom row) at 680 nm based on NA 0.2 illumination and NA 0.45 DF collection. The incident polarization is circular, and the intensity recorded with the QWP-LP pair for perpendicular (⊥) and parallel (//) components (left and right column, respectively). For n-SiO_2@Ag, the // component dominates, while for n-SiO_2@EA/Ag, the ⊥ component dominates. All images are recorded over the same location of the glass slide on which the microspheres have been dispersed. (All images horizontal size = 100 μm).

References

1. Lin, J.; Rodríguez-Herrera, O. G.; Kenny, F.; Lara, D.; Dainty, J. C., Fast vectorial calculation of the volumetric focused field distribution by using a three-dimensional Fourier transform. *Opt. Express* **2012**, *20*, 1060.
